# The Building Blocks of Integrated Care

**DOI:** 10.5334/ijic.2527

**Published:** 2016-10-28

**Authors:** Axel Kaehne

**Affiliations:** Faculty Health and Social Care, Edge Hill University, St Helens Road, Ormskirk, L39 4QP, GB

This special issue grows out of a collaborative project between IFIC and Edge Hill University (UK) that led to six webinars delivered by international experts on integrated care.^1^ The papers are reflection pieces by those who delivered the webinars providing a unique summary of accumulated expertise in some of the most important themes in the integrated care field. This special issue also offers a good opportunity to reflect on progress and challenges in the field as care integration increasingly gains policy support and gathers momentum in many countries across the world.

Integration of health and social care did not develop from a blank sheet. It grew out of long-standing concerns about care fragmentation and drew on a distinguished corpus of research on partnership and multi-agency collaboration in health and social care. Integration, just like its predecessor ‘partnerships’, carries largely positive connotations and thus quickly won support from policy makers resulting in a plethora of guidance documents and policy announcements. Despite the receptive wider context, integration remains a challenge for professionals and practitioners across the health and social care divide and the papers in this special issue address some of the most difficult barriers to implementing integrated care services.

Although integrating health and social care services is nothing if not popular amongst policy makers, it is a field where success remains isolated, incidental and highly dependent on contingent factors, such as effective local leadership and transformational synergies across sectoral divides. There is widespread agreement however on the need for change brought on by a long term shift from acute episodic health needs to care needs around chronic conditions requiring collaborative practices across the care continuum. As people live longer, health care systems are increasingly burdened by rising costs due to complex care needs that defy the traditional sector fault line between health and social care.

Yet despite the consensus for positive change, it is this fault line that remains stubbornly resistant to change in the face of considerable research evidence. The reasons are multi-faceted. So-called barrier research has long identified professional and organisational cultures, values and practices as significant challenges to forging truly co-ordinated responses to complex care needs of patients. Again, scholars of integrated care can draw on a rich pool of empirical evidence originating in singular intervention sites addressing these barriers. Solutions range from boundary spanners to full organisational integration pilots in some areas. Yet, none of these local solutions have gained traction or widespread adoption outside their locales for a variety of reasons. In fact, a sober assessment of the most successful pilots of integrated care (Kinzigtal, Kaiser Permanente) may reveal that they are unlikely to be a panacea for care fragmentation, but rather illustrative of a spectrum of solutions with variable applicability in specific local conditions.

As the papers in this special issue demonstrate, there are however some issues that are key to integrating care services, in a sense providing the foundations for integration success. One important aspect is training and education as part of workforce development. As medical education, nursing and social care vocational training remain strictly separated in most countries and higher education instruction in these fields is based on largely distinct models, practices, approaches and systems, staff development gains a critical role in fostering a collaborative climate for social care and medical staff to improve patient care quality and care outcomes. As we recognise that assessments, eligibility and care approaches may remain defined by largely different philosophies and payment systems, hopes for large scale systemic change may give way to realism. This realism may however succeed in focusing our attention to the things that are susceptible to change, incremental or gradual as it may be. Post-educational training and staff development then becomes a critical transformative catalyst, something increasingly acknowledged by managers in the health and social care sector.

Realistic expectations may also help us re-define the research challenge around integrated care. As a glance at the most prominent publications in the field easily show, research in integrated care remains highly conceptual, firmly focussed on process issues, and poorly evidenced for patient outcomes. It also often offers findings that are at best highly specific to local contexts with few generalisable insights. This means that, whilst we undoubtedly made enormous progress with process issues (broadly circumscribed by health management studies), we still remain at sea when it comes to identifying predictors of success for care integration with regard to patient outcomes. And, struggling to sufficiently evidence the link between integration processes and patient outcomes has a knock-on effect on the efforts of strategic and operational staff in care organisations trying to replicate success from other locations.

There are good reasons for this difficulty in knowledge transfer. Integration of care services deals with human beings and their social interactions. What works or does not work in integrating health and social care is characterised by high levels of contingency, specificity of contexts and extraneous factors that are difficult to quantify within conventional research designs. Although there are newly emerging paradigms that may go some way in addressing the issue of context-dependability and specificity, such as realist evaluations, we may also have to face the possibility that the most promising road to improving patient outcomes may remain under-evidenced.

The papers in this special issue provide valuable insights from international experts on themes of their expertise. They present the readers with an important contribution to the debate about the current direction of research and reveal what remains to be done. Nick Goodwin summarises the enormous amount of conceptual work that has been done in the field of integrated care in the last two decades. Victoria Stein tackles the issues of competencies and workforce development and how this can assist integrated care delivery. Robin Miller provides important insights into the barriers to integrated care delivery and the roles played by professional cultures and values. Talking about Scotland, Anne Hendry gives the readers a unique glimpse of an actual policy context in which health and social care integration may flourish. The papers by Apostolos Tsiachristas and Jon Glasby round up this special issue with critical comments on the building blocs of integrated care and reflections on the future of the field.

11 Sept 2016.

## Footnotes

http://integratedcarefoundation.org/events/webinar-series-the-building-blocks-of-integrated-care

